# Anti-Vimentin Nanobody Decreases Glioblastoma Cell Invasion In Vitro and In Vivo

**DOI:** 10.3390/cancers15030573

**Published:** 2023-01-17

**Authors:** Alja Zottel, Metka Novak, Neja Šamec, Bernarda Majc, Sara Colja, Mojca Katrašnik, Miloš Vittori, Barbara Hrastar, Ana Rotter, Andrej Porčnik, Tamara Lah Turnšek, Radovan Komel, Barbara Breznik, Ivana Jovčevska

**Affiliations:** 1 Medical Centre for Molecular Biology, Institute of Biochemistry and Molecular Genetics, Faculty of Medicine, University of Ljubljana, 1000 Ljubljana, Slovenia; 2 Department of Genetic Toxicology and Cancer Biology, National Institute of Biology, 1000 Ljubljana, Slovenia; 3 Jožef Stefan International Postgraduate School, 1000 Ljubljana, Slovenia; 4 Department of Biology, Biotechnical Faculty, University of Ljubljana, 1000 Ljubljana, Slovenia; 5 Department of Neurosurgery, University Medical Centre Ljubljana, 1000 Ljubljana, Slovenia; 6 Faculty of Chemistry and Chemical Technology, University of Ljubljana, 1000 Ljubljana, Slovenia

**Keywords:** glioblastoma, vimentin, nanobody, invasion, EMT

## Abstract

**Simple Summary:**

Glioblastoma is extremely lethal brain cancer. In our research, we investigated the effect of anti-vimentin nanobody Nb79 on glioblastoma cell invasion. The expression of vimentin in glioma tissues and cells was determined by RT-qPCR. Invasion assay was performed on differentiated glioblastoma cell line U-87 MG and stem cell line NCH421k in vitro and in vivo in zebrafish embryos. The effect of Nb79 on expression of EMT biomarkers was determined by Western blot and immunocytochemistry. Our study shows that vimentin is upregulated in glioblastoma tissue compared to lower grade glioma and non-tumour brain tissue. Nb79 reduced glioblastoma cell invasion by up to 64% in vitro and up to 21% in vivo. The tight junction protein ZO-1 had higher expression on the cell membrane, when treated with Nb79 compared to control. In conclusion, our results suggest that anti-vimentin nanobody is a promising tool to target glioblastoma cell invasion.

**Abstract:**

Purpose: Glioblastoma (GBM) is the most common primary brain tumour and one of the deadliest cancers. In addition to late diagnosis and inadequate treatment, the extremely low survival rate is also due to the lack of appropriate therapeutic biomarkers and corresponding therapeutic agents. One of the potential therapeutic biomarkers is the intermediate filament vimentin, which is associated with epithelial-mesenchymal transition (EMT). The purpose of this study was to analyse the effect of the anti-vimentin nanobody Nb79 on cell invasion in vitro and in vivo. To further our understanding of the mechanism of action, we investigated the association between Nb79 and EMT in GBM and GBM stem cells by analysing the expression levels of key EMT-related proteins. Methods: The expression of vimentin in glioma tissues and cells was determined by RT-qPCR. An invasion assay was performed on differentiated glioblastoma cell line U-87 MG and stem cell line NCH421k in vitro as well as in vivo in zebrafish embryos. The effect of Nb79 on expression of EMT biomarkers beta-catenin, vimentin, ZEB-1 and ZO1 was determined by Western blot and immunocytochemistry. Results: Our study shows that vimentin is upregulated in glioblastoma tissue compared to lower grade glioma and non-tumour brain tissue. We demonstrated that treatment with Nb79 reduced glioblastoma cell invasion by up to 64% in vitro and up to 21% in vivo. In addition, we found that the tight junction protein ZO-1 had higher expression on the cell membrane, when treated with inhibitory anti-vimentin Nb79 compared to control. Conclusion: In conclusion, our results suggest that anti-vimentin nanobody Nb79 is a promising tool to target glioblastoma cell invasion.

## 1. Introduction

Glioblastoma (GBM) is the most common and aggressive primary brain tumour [1], accounting for approximately 46% of all malignant brain and central nervous system tumours [2]. It is mainly diagnosed in the elderly, usually around the age of 64 years [3]. The five-year survival rate of GBM is particularly low, only 5.1%, but is usually higher in young adults and children [2]. Based on its histopathologic features, glioblastoma is considered as the most aggressive glioma type according to the World Health Organisation classification [1,4,5]. Histological features of GBM include extensive mitotic activity, vascular proliferation, necrosis, and infiltrative tumour nature. GBM is a highly invasive tumour with pleomorphic cells that do not metastasize to other organs of the body [6]. The only currently known risk factors for GBM are older age, genetics, radiation exposure, and environmental pollution [7]. Standard therapy for GBM includes complete surgical resection in combination with radiation therapy and chemotherapy with temozolomide (TMZ) [8]. Other treatments include bevacizumab, lomustine or carmustine and regorafenib [9]. Newer therapies such as tumour treating fields and immunotherapeutic approaches also emerged or are under intense investigation [8,10]. Glioblastomas exhibit high inter- and intra-tumoural heterogeneity. There are several proposed classifications of GBM. According to Verhaak et al., GBM is classified into four subgroups: proneural, neural, classical, and mesenchymal [11]. In 2017, Wang et al. proposed a classification of GBM into three subtypes: classical, proneural, and mesenchymal [12]. The malignant tumour is composed of various cells, including glioblastoma stem cells (GSCs), which play an important role in tumour growth and resistance to therapy. They are distinct from their differentiated tumour progeny, which means that a single therapy cannot be universally effective against all GBM cells [13].

One of the hallmarks of GBM is also infiltration into the brain parenchyma, for example along blood vessels, white matter, or the subarachnoid space [14]. The prominent event that promotes cell invasion and migration is the process of epithelial-mesenchymal transition (EMT), in which cells lose the epithelial-like phenotype and acquire mesenchymal characteristics that allow cell invasion [14]. Glioblastoma does not have an epithelial origin, but processes that mimic EMT are present in GBM and are associated with increased cell infiltration [15]. The transcription factors involved in EMT-like processes in GBM are similar to those in carcinomas, i.e., Slug, Snail1 and Snail2, Zeb1 and Zeb2 and Twist [14]. Gene silencing of SNAIL reduces GBM cell invasion, migration and proliferation, while ZEB1 and ZEB2 promote the upregulation of proteins related to the mesenchymal phenotype, such as N-cadherin, vimentin and fibronectin, and the downregulation of proteins related to the epithelial phenotype e.g., E-cadherin, claudins, occludins and cytokeratin [16]. These EMT transcription factors correlate with the invasive phenotype, tumour grade, treatment resistance, and poor survival of patients with GBM. Taken together, the EMT-like processes in GBM lead to the invasive mesenchymal GBM phenotype, although the signals controlling this process are less clear than in epithelial carcinomas. Cells with mesenchymal phenotype are nonpolar and loosely attached to the extracellular matrix [16]. One of the most important markers of the mesenchymal phenotype is vimentin, a type lll intermediate filament that has an important role in cancer invasion and metastasis [17]. Vimentin is also abundantly expressed in GBM [18], with higher expression being associated with shorter overall survival and shorter progression-free survival [19].

In this study, we focus on the effects of the previously developed anti-vimentin nanobody Nb79 [20], which is a nano biodrug that affects cell invasion. Nanobody is a part of heavy-chain only antibody, that is produced by only a few animals such as camelids and sharks [21,22]. As the name suggests, they are very small, 2 nm × 4 nm × 3.5 nm, and represent the smallest naturally occurring antibody unit that recognises antigen [23]. Structurally, they are similar to VH part of classical antibodies and consist of four framework regions and three antigen-binding loops [24]. Compared to classical antibodies, nanobodies have several advantageous properties, such as their small size which allows good penetration into solid tumours, their high affinity and stability, their straightforward production in *E. coli* or *S. cerevisiae* [23,25,26,27,28,29]. They are also more efficient in tumour penetration and may cross the blood-brain barrier, enabling treatment of central nervous system diseases [25,26,27]. They have high affinity, reaching the pM range, and can reach hidden epitopes of target proteins due to the long and flexible CDR3 loop [23]. The aim of this research was to examine the effect of the anti-vimentin nanobody Nb79 on the invasion of a glioblastoma differentiated cell line as well as glioblastoma stem cell (GSC) line in vitro as well as in vivo in zebrafish embryos. To further our understanding of the effect of this nanobody, we also examined the protein expression of common EMT biomarkers after cell treatment with Nb79 using Western blot and immunocytochemistry.

## 2. Materials and Methods

### 2.1. Nanobody Large-Scale Expression

Nanobody Nb79 was produced following the previously described protocols [30]. Briefly, nanobody was produced in *E. coli* and contained a 6 × His-tag. It was purified with nickel immobilised metal affinity chromatography and size exclusion chromatography. Nanobody was previously identified by Jovčevska et al. [20].

### 2.2. Cell Lines

Cell line U-87 MG was obtained from American Type Culture Collection (ATCC) and was cultured in high glucose DMEM supplemented with 10% fetal bovine serum (FBS), 2 mM L-glutamine and 1% penicillin/streptomycin. Glioblastoma stem cell line NCH421k was purchased from Cell Lines Service (CLS) GmbH and cultured in Neurobasal Medium (Invitrogen, Waltham, MA, USA) supplemented with 2 mM L-glutamine, 1 × B-27 supplement (Invitrogen), 1 U/mL heparin (Sigma-Aldrich, St. Louis, MO, USA), 20 ng/mL bFGF and EGF (both from Invitrogen, Life Technologies, Carlsbad, CA, USA) and 1% penicillin/streptomycin. For in vivo assay, cells were transfected with plasmid vectors pCMVDsRed-Express2 and pEGFP-N1 to stably express red fluorescent protein DsRed and green fluorescent protein eGFP, as described previously [31,32]. All cell lines were cultured at 37 °C, 5% CO_2_ and 95% humidity and were tested for mycoplasma contamination using the MycoAlert Mycoplasma Detection Kit (Lonza, Basel, Switzerland).

### 2.3. Establishment of Primary Glioblastoma and Glioblastoma Stem Cells

Primary glioblastoma cells were generated as described previously [33,34,35]. Briefly, fresh GBM tumour tissue biopsies were minced with scalpels in high-glucose Dulbecco’s modified Eagle’s medium (DMEM) (Hyclone, GE Healthcare, Chicago, IL, USA) supplemented with 10% FBS (Gibco, Thermo Fisher Scientific, Waltham, MA, USA), 2 mM L-glutamine, and 1% penicillin/streptomycin (both: Sigma-Aldrich, St. Louis, MO, USA) and seeded in culture flasks to culture the primary GBM cells. To obtain GSCs, GBM tumour tissue pieces were digested in a digestion buffer (200 U/mL collagenase II and IV (both: Gibco) in Neurobasal Medium. Cell suspensions were filtered using a cell strainer and resuspended in complete Neurobasal Medium. GSCs were propagated as floating spheres.

### 2.4. Tissues

Tissue biopsies were collected from patients who were operated on at the Department of Neurosurgery, University Medical Centre Ljubljana, Slovenia. Altogether, one-hundred and twenty-nine de novo glioblastoma, six recurrent glioblastoma and sixteen lower-grade glioma tissues were obtained. Tumours were diagnosed using the standard histopathology protocols at the Institute of Pathology of the Faculty of Medicine, the University of Ljubljana. As a reference, we also obtained 15 samples of non-cancerous brain tissues. Samples were snap-frozen and stored in liquid nitrogen for subsequent RNA isolation and protein extraction.

### 2.5. qPCR

Total RNA from the tissues and cells samples was isolated with AllPrep DNA/RNA/Protein Mini Kit (Qiagen, Germantown, MD, USA) following manufacturer’s protocol. Using High-Capacity cDNA Reverse Transcription Kit (Thermo Fischer Scientific, Waltham, MA, USA), 1 μg of RNA was transcribed to cDNA. Next, qPCR was performed with Fluidigm BioMark HD System Real-Time PCR (Fluidigm) using 48.48 Dynamic Arrays IF (Fluidigm Corpora- 463 tion, San Francisco, CA, USA), where 42 samples and 24 assays (probes) were mixed pairwise in nanoliter chambers to enable parallel analysis of 2304 reactions. Analysis and visualisation of results were performed with Fluidigm real time qPCR analysis software and quantGenius software [36]. Results were normalised to two housekeeping genes, *HPRT1* and *GAPDH*. Statistical analyses were performed with one-way ANOVA in GraphPad Prism (GraphPad Software Inc., La Jolla, CA, USA).

### 2.6. Invasion Assay

Invasion of U-87 MG and NCH421k was determined as described before [33]. We used 24-well Transwell units with 6.5 mm inserts and 8 μm pores (Corning, New York, NY, USA). For the assay 0.5 mg/mL Matrigel (Becton Dickinson, Franklin Lakes, NJ, USA) in DMEM without FBS was added to the upper container. After 30 min, 10,000 U-87 MG cells/insert and 80,000 NCH421k cells/insert were added. The lower chamber was filled with DMEM with 10% FBS. Nanobody Nb79 in a final concentration 100 µg/mL was added to the upper chamber. Cells were incubated for 48 h at 37 °C in 5% CO_2_. Cells were removed from the upper part of the chamber, which was then washed twice with PBS. Cells on the lower side of the chamber were fixed with 4% paraformaldehyde for 15 min. Afterwards, insert was washed twice with PBS. Invaded cells were then stained with 0.1% crystal violet for 10–15 min and counted using the Nikon Eclipse Ti-inverted microscope (Nikon Instruments, Melville, NY, USA) at 4× magnification.

### 2.7. Zebrafish Embryo Model

The experiment was performed as described by Porčnik et al. [35]. Briefly, embryos of wild-type AB zebrafish (*Danio rerio*) were maintained at 28 °C and injected with 50–100 fluorescent GBM cells (U87dsRED and NCH421keGFP) in presence of 1.4 mg/mL Nb79 at 52 h after fertilization using MICROINJECTOR system (Tritech Research, Los Angeles, CA, USA). After cell implantation, embryos were incubated in reconstituted water at 31 °C in 48-well plates and imaged with an Eclipse TE300 fluorescence inverted microscope (Nikon) at 24 h and 72 h after implantation in embryos lying on their sides. As the control, cells, U87dsRED and NCH421keGFP, without Nb79 were injected. Invasion of GBM cells from glioblastoma xenografts was measured in images of embryos in lateral orientation, as described previously [31]. Differences in relative cell invasion were determined by measuring tumour length measurements at 24 h and 72 h after implantation using ImageJ software (Software for image analysis, National Institutes of Health, Bethesda, MD, USA, http://rsbweb.nih.gov/ij/, accessed on 22 August 2021). The quantification of results is described in [35]. Mann–Whitney test was used for statistical analysis.

### 2.8. Western Blot

Initially, NCH421k cells (6 × 10^5^) and U-87 MG (5 × 10^5^) were seeded in wells of six-well plate and incubated for 24 h. Next day, anti-vimentin (Nb79) nanobody was added to final concentration 100 μg/mL and incubated for 48 h. After incubation, cells were washed with PBS three times and treated with 50 μL of RIPA puffer (ThermoFisher, Waltham, MA, USA) together with Halt protease inhibitors (ThermoFisher) and phosphatase inhibitors (ThermoFisher) for 15 min to enable cell lysis. Afterward, samples were collected to a tube and centrifuged for 15 min at 13,000 rpm. Supernatant was transferred to new microcentrifuge tubes and concentration of each cell lysate was measured on Synergy H4 Hybrid Multi-Mode Microplate Reader (BioTek, Winooski, VT, USA) using BCA Protein Assay Kit (ThermoFisher Scientific). For Western blotting, 10 μg of each sample was separated using NuPAGE 4% to 12% Bis-Tris Mini Gels (Invitrogen) and transferred to an Immobilon-P Transfer Membrane (Milipore, Burlington, MA, USA). Membrane was then blocked with 5% milk in PBS for 1 h at room temperature, and incubated with primary antibodies overnight at 4 °C. The antibodies and their working dilutions (all from Cell Signaling Technology, Danvers, MA, USA) were 1:2000 rabbit anti-ZO-1 (cat. number: 8193), 1:2000 rabbit anti-beta-catenin (cat. number: 8480), 1:2000 rabbit anti-vimentin (cat. number: 5741), 1:2000 rabbit anti-ZEB1 (cat. number: 3396). The next day, membrane was incubated with 1:5000 secondary anti-rabbit antibodies IgG, HRP-linked Antibody (cat. number: 7074, Cell Signaling Technology) for 2 h at room temperature. As a loading control, we used GAPDH (0.05 μg/mL, cat. number G8795 Sigma Aldrich, St. Louis, MO, USA). Signals were detected using SuperSignal West Pico Chemiluminescent Substrate (Thermo Scientific) and Fujifilm LAS-4000 (Tokyo, Japan). The results were analysed with the Multi Gauge version 3.2 software (FUJIFILM Manufacturing Corp., Tokyo, Japan). All the whole western blot figures can be found in the Supplementary Materials.

### 2.9. Immunofluorescence

To analyse the expression of specific EMT biomarkers and determine their cellular location, we performed immunocytochemistry on GBM cells. Total of 1.5 × 10^5^ U-87 MG cells were seeded on poly-d-lysine pre-treated 12-well plate and incubated for 24 h. Next day, 100 μg of Nb79 was added to the cells and incubated for 48 h. For control, cells without nanobody were used. After two days, cells were washed three times with PBS and fixed with ice-cold 4% formaldehyde for 15 min at room temperature. Cells were washed three times for 10 min with PBS and permeabilised with 0.1% Triton X-100 in PBS for 15 min at room temperature. Next, cells were blocked with 5% bovine serum albumin in PBS for 1 h at room temperature. Primary antibodies (Cell Signaling Technology) were then added at appropriate dilutions (1:200 rabbit anti-vimentin cat. number 5741, 1:100 rabbit anti-ZEB1 cat. number 3396, 1:100 rabbit anti-beta-catenin cat. number 8480, 1:100 rabbit anti ZO-1 cat. number 8193) and incubated overnight at 4 °C. Next day, cells were washed three times with PBS for 10 min, followed by 1 h incubation with secondary antibodies (anti-rabbit 1:1000 with CF640 fluorophore, Sigma Aldrich, cat. number SAB4600399) at room temperature. An additional control of cells with secondary antibody (without the previous addition of primary antibody) was applied. Afterwards, cells were washed three times with PBS for 10 min and incubated with 4′,6-diamidino-2-phenylindole dihydrochloride (DAPI) for 10 min. Subsequently, the cells were washed three times with PBS for 10 min and mounted on slides using ProlongGlass (ThermoFisher Scientific). Samples were observed under microscope Axio Imager M2 (Zeiss, Jena, Germany) equipped with ZEN software under the 63× oil-immersion objective (Plan-Apochromat 63×/1.40 Oil DIC M27). They were excited by filters at wavelengths of 335–383 nm (DAPI) and 574–599 nm (rhodamine-based far-red fluorescent dye (CF640R)), with filter emission wavelengths of 420–470 nm (DAPI) and 612–682 nm (CF640R). The images were analysed with ImageJ.

## 3. Results

### 3.1. Vimentin Gene Expression in GBM Tissues and Cells

**Figure 1:**
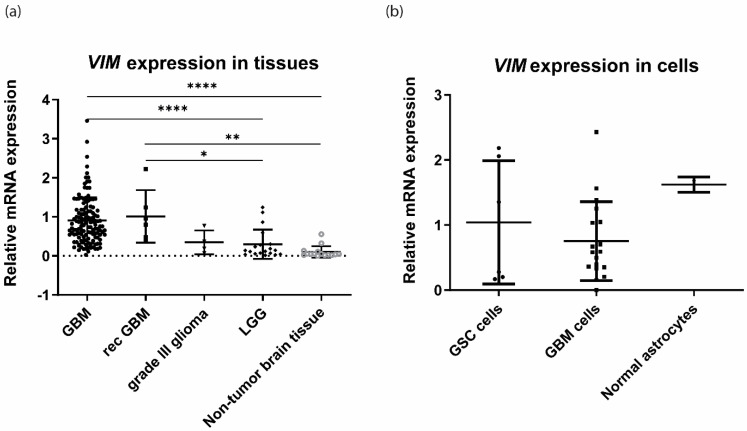
Relative VIM mRNA expression of different glioma grades recurrent GBM and reference brain tissues, and glioblastoma cells, stem cells and reference astrocytes: (**a**) The expression of VIM in GBM, recurrent GBM (rec GBM), grade lll glioma, lower-grade glioma (LGG) and non-tumour brain tissue. N (GBM tissue) = 129, N (rec GBM) = 6, N (grade lll glioma) = 4, N (LGG) = 16, N (normal) = 15. (**b**) The expression of VIM in glioblastoma stem cells (GSC), GBM cells and non-malignant astrocytes. N (GSC cells) = 6, N (GBM cells) = 18, N (normal astrocytes) = 2. All the data in a. and b. present means +/− SD. One-way ANOVA statistical test was used for analyses. * *p* < 0.05, ** *p* < 0.01, **** *p* < 0.0001.

First, we analysed the expression of vimentin mRNA (*VIM*) in different glioma tumours (Figure 1a). The tissues that were included in the study were GBM, recurrent GBM, grade III glioma, and lower-grade glioma (LGG). Non-tumour brain tissue (normal) was used as a reference. The results showed that the expression of *VIM* in GBM and rec GBM was significantly higher compared to LGG and normal brain tissue. There were no differences between grade lll glioma and other glioma tumours analysed. Nevertheless, the expression level between grade lll glioma and LGG was similar, suggesting that higher *VIM* expression is related to the most aggressive glioma type i.e., GBM.

The expression of *VIM* was also analysed on cellular level (Figure 1b) in glioblastoma stem cells (GSCs), glioblastoma cells (GBM cells) and normal human astrocytes. The results show that there were no differences in *VIM* expression observed among analysed cell types.

### 3.2. Anti-Vimentin Nanobody Nb79 Decreases GBM Cell Invasion In Vitro and In Vivo

**Figure 2:**
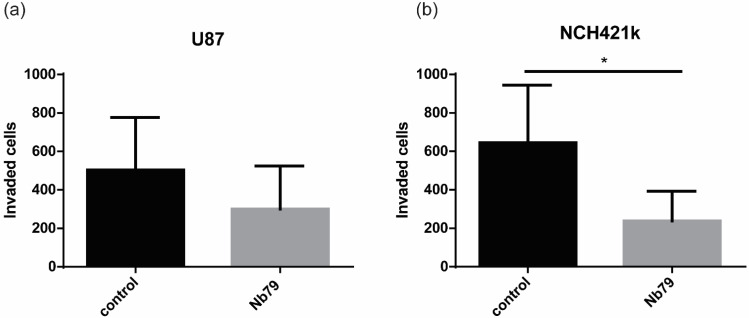
The effect of Nb79 nanobody on tumour cell invasion. The effect on cell invasion was determined in U87DsRed (**a**) and NCH421keGFP (**b**) cells. Nanobody at concentration 100 μg/mL was added. Nb79 inhibited the invasion of both U-87 MG and NCH421k cells. As a control we used cells without added nanobody. Unpaired *t*-test was used for statistical analysis. Experiment was performed in 6 replicates. Data show means +/− SD. * *p* < 0.05.

The significant overexpression of *VIM* in GBM tissues indicates that it is a potential and suitable biomarker candidate for GBM targeting. We then determined the effect of Nb79 on cell invasion. In vitro cell invasion assay was performed on differentiated GBM cell line U-87 MG (Figure 2a) and glioblastoma stem cell line NCH421k (Figure 2b) using Transwell invasion assay with Matrigel. The results show that Nb79 decreased the invasion of both cell lines analysed, U87 MG and NCH421k, for 40% and 64%, respectively.

**Figure 3:**
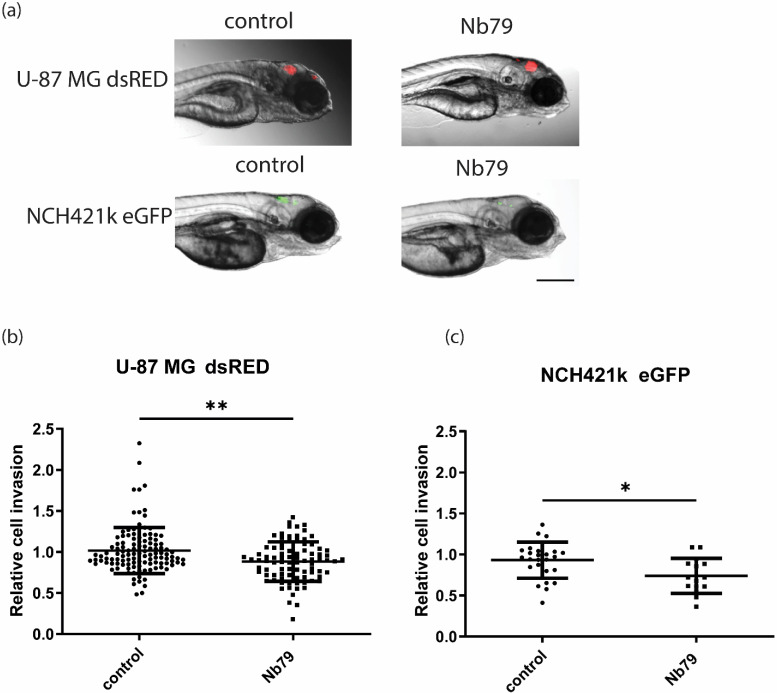
The effect of anti-vimentin Nb79 nanobody on invasion of GBM cells in Zebrafish model. (**a**) Embryos with implanted U-87 MG and NCH421k are shown under fluorescence microscope at 4× magnification. The effect on invasion was determined on U-87 MG (**b**) and NCH421k (**c**) cells in presence or absence (control) of 1.4 mg/mL Nb79. Each dot represents one embryo. For U-87 MG 92–116 embryos were used and for NCH421k 14–26. Data are shown as means ± SEM. Relative tumour size represents tumour size after 72 h normalised to tumour size after 24 h after injection. Statistical analysis was performed using Mann–Whitney test. * *p* < 0.05, ** *p* < 0.01. Scale bar = 300 μm.

Next, we analysed whether the in vitro anti-invasion effect can also be observed in zebrafish embryos in vivo. For this purpose, U-87 MG and NCH421k cells were injected in brains of zebrafish embryos in the presence of Nb79 as described in Section 2. Relative cell invasion was calculated as a change in tumour length between 24 h and 72 h after xenotransplantation. Our results show that the anti-invasion effect is also observed in animal model, as the invasion of U-87 MG decreased by 13% (Figure 3a,b) and the invasion of NCH421k by 21% (Figure 3a,c), both significantly. Tumour length was determined to measure cell invasion because it is sensitive to cell dispersion, i.e., cell movement or invasion. Since an increase in tumour length may also result from proliferation, we combined these results with those of tumour growth, determined as a change in mean fluorescence intensity of GBM xenografts, to determine if this was the case. Since GBM xenograft growth was not altered in the presence of nanobody (Supplementary Figure S1), we concluded that nanobody decreased only GBM cell invasion.

### 3.3. Anti-Vimentin Nanobody Nb79 Affects the Location of Epithelial to Mesenchymal Transition (EMT) Markers

**Figure 4:**
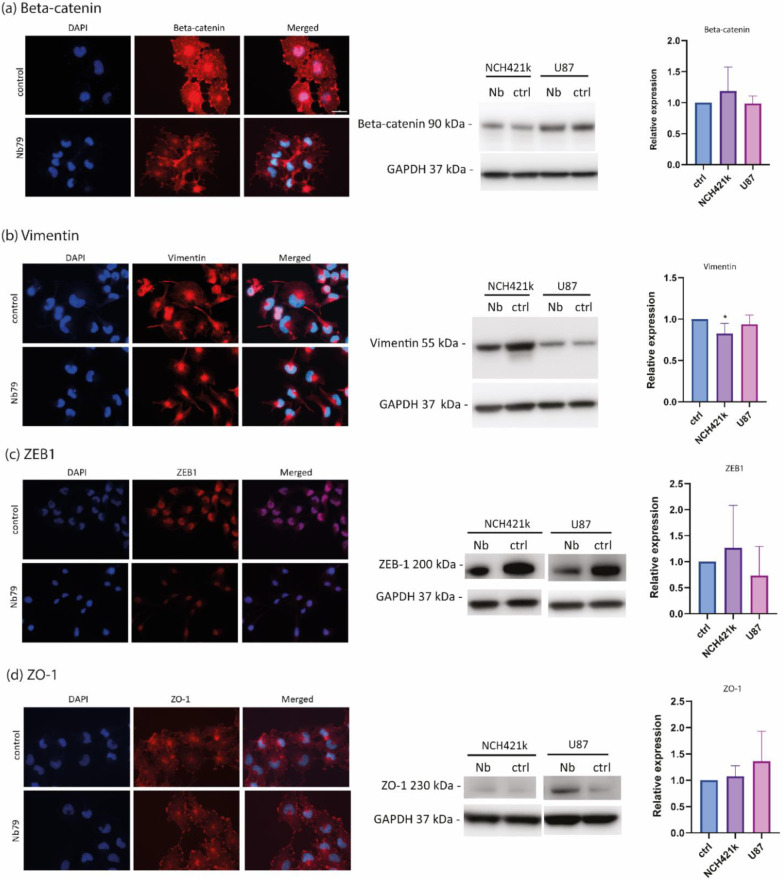
The effect of Nb79 on expression and localisation of EMT biomarkers determined by immunocytochemistry and Western blot. Experiment was performed on U-87 MG for immunocytochemistry and U-87 and NCH421k for Western blot. Cells were treated with 100 μg/mL of Nb79 for 48 h. Cells without addition of Nb79 were used as a control. Markers beta-catenin (**a**), vimentin (**b**), ZEB1 (**c**), and ZO-1 (**d**) were detected. For immunocytochemistry, cells were stained with suitable commercial primary and secondary antibodies conjugated with CF640R (red) and cell nuclei were stained with DAPI (blue). Scale bare of 20 μm is shown as a white line on top right picture and is applied to all of the images. For Western blot data analysis (right graphs) was performed. Data show means ± SD. * *p* < 0.05. At least three independent experiments were performed. Raw images of one of the replicates is presented in Supplementary Figure S2. Results were analysed with One-way ANOVA. All the whole western blot figures can be found in the Supplementary Materials.

We have demonstrated that anti-vimentin Nb79 has anti-invasion effect in vitro as well as in vivo. To deepen our understanding of mechanisms behind Nb79 function, we analysed the protein expression levels of key protein biomarkers of EMT, such as beta-catenin, vimentin, ZEB1 and ZO-1. The protein expression levels were determined by Western blot and changes in protein localisation were analysed with immunocytochemistry (Figure 4).

No statistical differences were observed in beta-catenin, ZEB1 and ZO-1 expression level between control and Nb79. After the addition of Nb79, beta-catenin signal in nucleus and cytoplasm decreased compared to the control. Moreover, the signal was stronger in cell contacts (Figure 4a). When Nb79 was added, the protein expression level of vimentin in NCH421k decreased while there were no differences observed in U-87 MG (Figure 4b). Immunofluorescence showed that the signal intensity of ZEB1 was considerably lower in U-87 MG when Nb79 was added (Figure 4c). Immunocytochemistry results also show that the signal of epithelial marker ZO-1 on the cell membrane is more pronounced when Nb79 was added compared to the control (Figure 4d). Overall, there were no or little differences detected with Western blotting.

## 4. Discussion

Glioblastoma (GBM) is the most common primary brain tumour with an exceptionally fatal prognosis, as most of the patients die within 14 to 16 months after diagnosis, despite conventional therapy (4). One of the major challenges is the lack of reliable therapeutic biomarkers and effective therapy. Our study was focused on vimentin, a potential GBM target of tumour invasion and spread. Vimentin is a cytoskeletal protein and belongs to type lll intermediate filaments [19]. In normal human brain, it is expressed in ependymal cells, the choroid plexus, meningeal cells, and some subpial cells, endothelial cells. It is also expressed in astrocytes, but with weaker intensity compared to glioblastoma cell lines [37,38]. In contrast, vimentin is highly expressed in GBM. Our results show that *VIM* gene expression is about 11-fold higher in GBM tissue compared with non-tumour brain tissues (Figure 1a) and was also significantly higher compared with LGG. However, there were no statistical differences in vimentin expression between glioblastoma differentiated cells, GSCs and astrocytes. High standard deviations of gene expression in GBM and GSC cells suggest that the cells are very heterogeneous in terms of VIM expression and cells isolated from certain patients have higher expression in glioblastoma cells compared with astrocytes. Protein expression analyses will be performed in further experiments to confirm results on protein level. In our previous research, we identified anti-vimentin nanobody, Nb79, as a potential nano-drug to target GBM cells [20]. Vimentin is a marker of mesenchymal phenotype and has been associated with tumour migration and invasion [18]. Recently, a pro-angiogenic and pro-inflammatory role of vimentin was discovered [39], suggesting that vimentin has various functions in tumours, including glioma progression. In the present study, we investigated the effect of anti-vimentin nanobody, Nb79, on GSCs, NCH421k and differentiated GBM cell line U-87 MG invasion in vitro as well as in vivo. The results show that Nb79 considerably decreased cell invasion in vitro and the effect was higher in NCH421k cells (64% decrease) compared to U-87 MG (40% decrease). A high anti-migratory effect of Nb79 was reported in our previous study [38], in which we also showed that it had no effect on normal astrocytes. Second, we also tested the effect of Nb79 in vivo in zebrafish embryos, using U-87 MG and NCH421k xenografts. Zebrafish are a particularly useful model in cancer research because the embryos are transparent, and the tumour can be analysed at the cellular level using a high-resolution fluorescence microscope [40,41,42,43]. Moreover, they are also low-cost, high-throughput models that allow the study of various cancer processes such as invasion, angiogenesis, and response to drugs. Moreover, zebrafish have orthologs for 82% of genes associated to human disease [42]. Our results from the cell invasion study in the zebrafish model showed that invasion decreased in both U-87 MG (for 13%) and NCH42k (for 21%) cell lines after Nb79 nanobody treatment. The effect was weaker compared to in vitro, which may be due to the presence of a tumour microenvironment that can alter the response of cells to the presence of nanobodies. In zebrafish embryos, GBM cell xenografts exhibit more 3D confirmation mimicking in vivo tumour architecture. Moreover, GBM cell xenografts are surrounded by zebrafish brain and endothelial cells and extracellular matrix that can affect GBM cell phenotype and consequently their response to nanobody [31,44,45]. Importantly, the invasion inhibitory effect was not related to cytotoxicity of tumour cells, as the relative tumour growth did not decrease when the nanobody was added. The systemic cytotoxicity of the nanobody was not assessed in this study because the nanobody was applied together with GBM cells into the embryo brain.

Finally, we aimed to further investigate the mechanisms behind the anti-invasion effect of Nb79. Therefore, we determined the protein expression levels of key markers of epithelial phenotype (ZO-1) and mesenchymal phenotype (ZEB1, beta-catenin, vimentin) in U-87 MG and NCH421k cells. ZO-1 is one of the scaffold proteins in the tight junctions and transduces signals to the cell interior [46]. Our immunostaining results show that there were no differences in signal intensity in U-87 MG, but the signal in the membrane was stronger in the presence of Nb79 than in the control. According to the UNIPROT, the cellular location of ZO-1 is the cell membrane [47]. During epithelial migration, ZO-1 delocalises from the membrane, and depending on the specific state of differentiation and migration, ZO-1 may be found in various cellular departments, including the nucleus, and acts as a signalling protein [48]. The expression of beta-catenin, a marker of the mesenchymal phenotype, was also studied. Our immunofluorescence results showed that the signal in the nucleus was more intense in the control sample compared with Nb79. A similar change was observed by Ding et al., who showed that knockdown of vimentin resulted in decreased expression of beta-catenin in the cytoplasm and the nucleus [49]. Beta-catenin is a structural components of cadherin-based adherens junctions and one of the key players in Wnt signalling [50]. It is highly expressed in brain tumours [51]. It is mainly localised on the cytoplasmic side of the membrane. During the EMT, levels of membrane E-cadherin decrease, beta-catenin is released into the cytoplasm, from where it can enter the nucleus to activate transcription of Wnt/β-catenin target genes [50]. The expression of ZEB1 protein, one of the major transcription factors regulating and promoting EMT [52], was decreased in U-87 MG in the presence of Nb79, which was confirmed by immunofluorescence. However, the differences observed on Western blot level were small. The mechanism of nanobody Nb79 is not yet known. It is possible that the nanobody binds to vimentin and prevents binding of other proteins. To clarify the role of Nb79, the binding domain would need to be determined. In addition, only a limited number of markers and only EMT pathway were tested in this study. As mentioned earlier, vimentin plays and important role in several pathways and to better understand the mechanism, the study could be expanded to include larger set of markers covering different pathways.

In summary, this is the first study to demonstrate that anti-vimentin nanobody reduces the invasion of glioblastoma cells, particularly GSCs that are resistant to existing therapies and are responsible for tumour recurrence [53]. This first preclinical study offers the possibility that nanobodies could be further tested in ex vivo GBM models that better recapitulate the tumour, such as glioblastoma-derived organoids or tissue-slice cultures, as well as mouse models.

## 5. Conclusions

In this study, we have shown that vimentin is overexpressed in glioblastoma tissue compared with lower grade gliomas and non-tumour brain tissue. The anti-vimentin nanobody Nb79 significantly reduced the invasion of the differentiated glioblastoma cell line U-87 MG and the glioblastoma stem cell line NCH421k, with greater effect in NCH421k compared with U-87 MG cells. The invasion inhibitory effect of Nb79 was also observed in vivo in zebrafish embryos, again with a greater effect on glioblastoma stem cells NCH421k. Treatment of U-87 cells with the nanobody also resulted in an increased presence of the epithelial marker ZO-1 on the cell membrane. In conclusion, Nb79 is a suitable molecular tool to target tumour cell invasion, the characteristic and lethal hallmark of GBM cells and GSCs.

## Data Availability

Research data is available at first and leading authors upon request.

## Supplementary Materials

The following supporting information can be downloaded at: https://www.mdpi.com/article/10.3390/cancers15030573/s1, Figure S1: In vivo imaging of tumor growth; Figure S2: Western blot raw images.
