# Supplementary material for: Anti-Vimentin Nanobody Decreases Glioblastoma Cell Invasion In Vitro and In Vivo
Source: Cancers (Basel). 2023 Jan 17;15(3):573. doi: 10.3390/cancers15030573 (PMC9913279; doi:10.3390/cancers15030573)
Supplement: Supplementary file 1 [file cancers-15-00573-s001.zip › Supplementary figure S1.pdf]

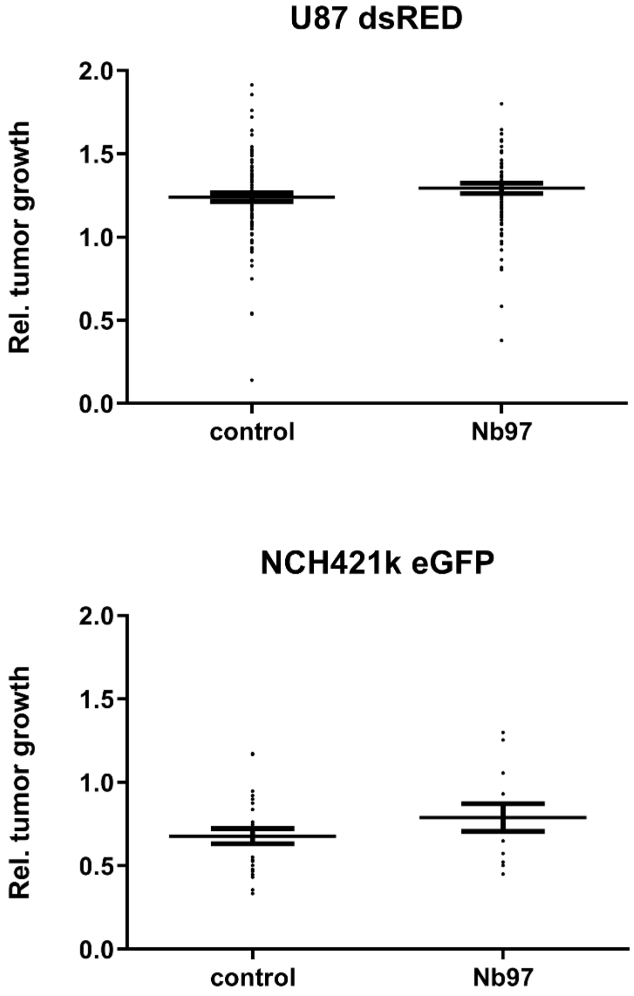

Supplementary Figure S1 *In vivo* imaging of tumour growth, determined as a change in mean fluorescence intensity of GBM xenografts, in the brain of zebrafish embryos**.** Embryos 72h after the xenotransplantation of GBM cells in the brain without (control) or with Nb97 nanobody. Data are shown as means ± S.D., dots on graphs represent each embryo. Fluorescence intensity of GB xenograft at 72h was normalized to fluorescence intensity of GBM xenograft at 24h after xenotransplantation to determine relative tumour growth. No statistically significant differences between the groups were observed.
