# Supplementary material for: Anti-Vimentin Nanobody Decreases Glioblastoma Cell Invasion In Vitro and In Vivo
Source: Cancers (Basel). 2023 Jan 17;15(3):573. doi: 10.3390/cancers15030573 (PMC9913279; doi:10.3390/cancers15030573)
Supplement: Supplementary file 1 [file cancers-15-00573-s001.zip › Supplementary figure S2.pdf]

1. **Beta-catenin**

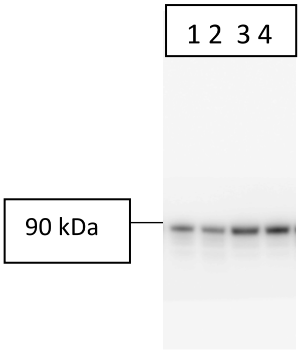

941	854	1485	1563

1 –NCH421k – Nb treatment
2 – NCH421k – control
3 –U87 – Nb treatment
4 – U87 - control

GAPDH

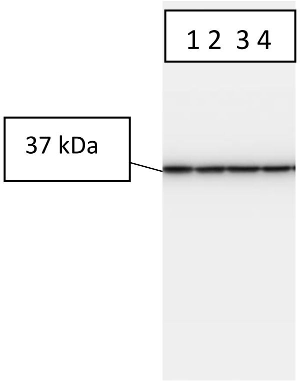

1739	1599	1529	1595

2. Vimentin

55 kDa

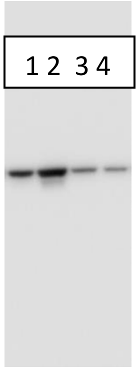

1 –NCH421k – Nb treatment
2 – NCH421k – control
3 –U87 – Nb treatment
4 – U87 - control

1487	1988	1006	794

GAPDH

37 kDa

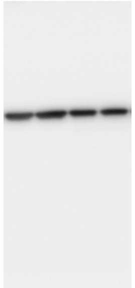

1396	1563	1429	1336

3. ZEB1

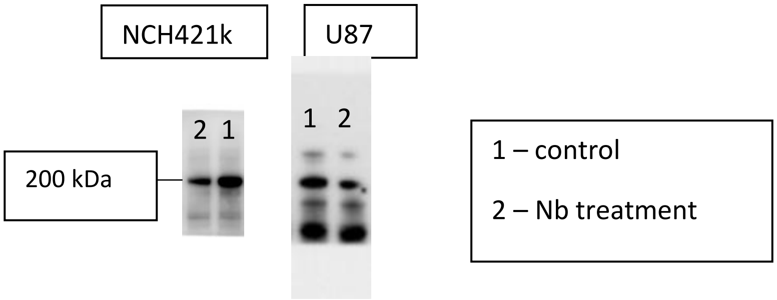

933	1302	1585	811

GAPDH

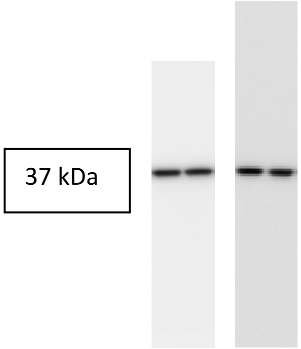

1561	1415	1226	1373

4. ZO1

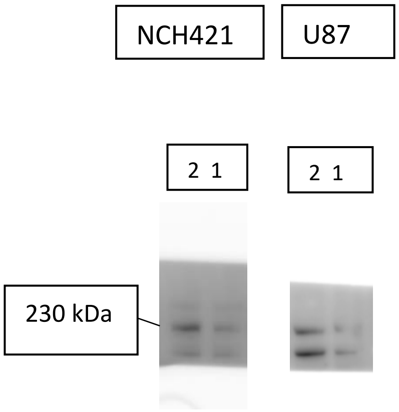

1 – control

2 – Nb treatment

2352	1013	3360	936

GAPDH

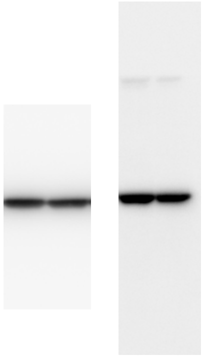

2869	1739	4697	4098
